# Benchmarking of five commercial deformable image registration algorithms for head and neck patients

**DOI:** 10.1120/jacmp.v17i3.5735

**Published:** 2016-05-08

**Authors:** Jason Pukala, Perry B. Johnson, Amish P. Shah, Katja M. Langen, Frank J. Bova, Robert J. Staton, Rafael R. Mañon, Patrick Kelly, Sanford L. Meeks

**Affiliations:** ^1^ Department of Radiation Oncology UF Health Cancer Center at Orlando Health Orlando FL USA; ^2^ Department of Radiation Oncology University of Miami Miami FL USA; ^3^ Department of Radiation Oncology University of Maryland Baltimore MD USA; ^4^ Department of Neurosurgery University of Florida Gainesville FL USA

**Keywords:** deformable image registration, virtual phantoms, quality assurance, adaptive radiotherapy, head and neck cancer

## Abstract

Benchmarking is a process in which standardized tests are used to assess system performance. The data produced in the process are important for comparative purposes, particularly when considering the implementation and quality assurance of DIR algorithms. In this work, five commercial DIR algorithms (MIM, Velocity, RayStation, Pinnacle, and Eclipse) were benchmarked using a set of 10 virtual phantoms. The phantoms were previously developed based on CT data collected from real head and neck patients. Each phantom includes a start of treatment CT dataset, an end of treatment CT dataset, and the ground‐truth deformation vector field (DVF) which links them together. These virtual phantoms were imported into the commercial systems and registered through a deformable process. The resulting DVFs were compared to the ground‐truth DVF to determine the target registration error (TRE) at every voxel within the image set. Real treatment plans were also recalculated on each end of treatment CT dataset and the dose transferred according to both the ground‐truth and test DVFs. Dosimetric changes were assessed, and TRE was correlated with changes in the DVH of individual structures. In the first part of the study, results show mean TRE on the order of 0.5 mm to 3 mm for all phantoms and ROIs. In certain instances, however, misregistrations were encountered which produced mean and max errors up to 6.8 mm and 22 mm, respectively. In the second part of the study, dosimetric error was found to be strongly correlated with TRE in the brainstem, but weakly correlated with TRE in the spinal cord. Several interesting cases were assessed which highlight the interplay between the direction and magnitude of TRE and the dose distribution, including the slope of dosimetric gradients and the distance to critical structures. This information can be used to help clinicians better implement and test their algorithms, and also understand the strengths and weaknesses of a dose adaptive approach.

PACS number(s): 87.57.nj, 87.55.dk, 87.55.Qr

## I. INTRODUCTION

Deformable image registration (DIR) is the nonaffine process of mapping voxels from one image to another where the individual vectors that describe the mapping may vary in both magnitude and direction from their neighbors. The entire process is encompassed by the deformation vector field (DVF), which aggregates the individual vectors into a single map and specifies the coordinate transformation between the two datasets. The DVF facilitates the transfer of information and allows the user to perform a number of useful functions such as contour propagation^(1)^ or dose accumulation.^(2)^ Initially, these functions were primarily limited to academic centers where many of the deformation algorithms were developed. Over the past several years, however, the use of DIR has expanded widely to the point where DIR is now included in several commercial treatment planning and contouring platforms. Commercial DIR algorithms are powerful and complex. Given two datasets with inherent differences, DIR algorithms are capable of quantifying these differences and minimizing them by creating a new deformed image, the result of a process which morphs one image into another. This ability to deform almost anything is also the fundamental limitation of these systems. Because DIR algorithms are based on complex mathematical models, there is no guarantee that the deformation defined by the DVF will represent biological change accurately. Thus, as with any new tool intended for use within the radiation oncology clinic, implementation must go hand‐in‐hand with validation.

Much research has been done in this area, primarily through the creation of ground‐truth models and QA metrics. In relation to the former, ground‐truth models consist of two image sets linked via a known DVF or transferred content such as known landmarks. In an effort to provide consistent datasets for algorithm validation, several researchers have made their ground‐truth models publicly available. Examples include the extended cardiac–torso (XCAT) phantom,^(3)^ the point‐validated pixel‐based breathing thorax model (POPI model),^(4)^ the DIR‐Lab Thoracic 4D CT model,^5^, ^6^ and those provided as part of the EMPIRE 10 challenge.^(7)^ In an effort to improve the correlation of computer‐based phantoms with the actual anatomical changes seen over a course of radiation therapy, the current authors previously developed synthetic datasets derived from clinical images of real head and neck patients.^(8)^ These phantoms are publicly available for users to download as part of the Deformable Image Registration Evaluation Project (DIREP) (http://sites.google.com/site/dirphantoms).

With each of the models described in the previous section, it is left to the clinical physicist to test their system, analyze the data, and compare their results with known benchmarks. Several metrics have been proposed for this analysis, ranging from the simple (DICE similarity) to the abstract (the Jacobian of the DVF). One commonly used metric is the target registration error (TRE), which describes the difference between co‐located voxels once they have been transferred through the ground‐truth DVF and a test DVF. In the upcoming report on DIR validation by AAPM's Task Group 132, the proposed goal (as opposed to “tolerance”) for TRE is 95% of voxels within 2 mm.^(9)^ One limitation shared by all DIR metrics, however, is the disconnect between the quantification of DIR error and the effect that error has on a given dose distribution. This is a complicated issue akin to relying on the gamma criterion for IMRT QA analysis. Currently, there are few publications which translate target registration error to DVH error, and thus one of the aims of this research was to investigate this process. In addition to TRE/DVH error, the traditional commissioning framework of test, analyze, and compare is hindered by the current lack of benchmarks specifically purposed for the clinical validation of commercial algorithms. Due to the fact that the vast majority of usage will be through one of these commercial platforms, it is important to provide clinical physicists comparison data which are directly linked to open‐source, ground‐truth models. In this way, end users can assess their own implementation using the same tools and metrics as those used during the benchmarking process.

In this work, the five most prevalent commercial DIR algorithms are assessed using the 10 phantom series of the DIREP ground‐truth model. Benchmark data are presented for each algorithm using TRE as the DIR metric. Questions relevant to the commissioning process are discussed, including what constitutes a DIR failure, what differences may be expected between different commercial algorithms, and where those differences occur. A further analysis is performed to translate TRE into DVH error, and the results are discussed in relation to the strengths and weakness of such an approach.

## II. MATERIALS AND METHODS

### A. Ground‐truth model

The Deformable Image Registration Evaluation Project (DIREP) includes a library of 10 virtual phantoms. These phantoms were previously created based on volumetric imaging of 10 head and neck cancer patients.^(8)^ Briefly, each patient received a start‐of‐treatment CT (SOT) and a near end‐of‐treatment CT (EOT). The SOT dataset for each patient was deformed in a forward fashion to represent the anatomy of the EOT dataset (i.e., the EOT was the target dataset and the SOT dataset was deformed to match it). This was done using a combination of a biomechanical algorithm^10^, ^11^ and human‐guided thin plate splines^(12)^ available as a deformation tool within the ImSimQA software package (Oncology Systems Limited, Shrewsbury, Shropshire, UK). These tools allowed for the modeling of head rotation and translation, mandible rotation, spine flexion, shoulder movement, hyoid movement, tumor/node shrinkage, weight loss, and parotid shrinkage.^(13)^ The combinative approach of utilizing multiple algorithms applied iteratively with human intervention minimizes the bias towards any particular algorithm during subsequent DIR using these phantoms.

**Figure 1:**
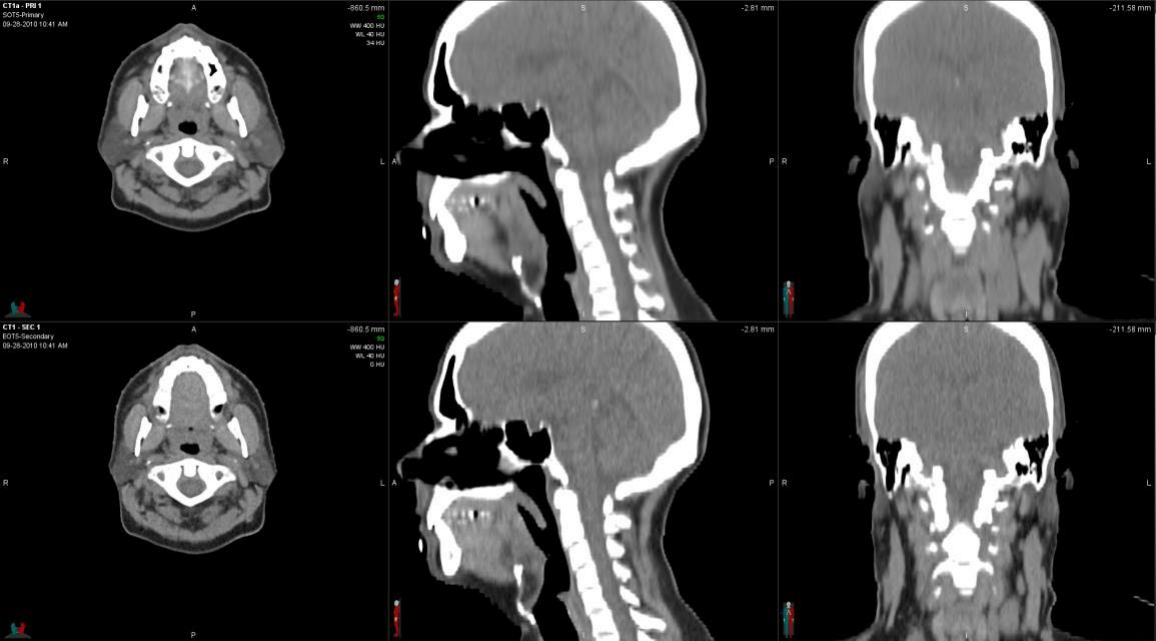
Virtual phantom example (Phantom 5). The top row shows the planning image set and the bottom row shows the simulated EOT image set. Comparison of the images reveals the simulated parotid shrinkage (axial view), head rotation and spine fexion (sagittal view), and weight loss (coronal view).

The result of the deformation process was a simulated EOT dataset for each patient where the ground‐truth deformation was known. Together, the SOT and simulated EOT images form a virtual phantom that may be imported into a third‐party DIR software package. The DVF from any third‐party algorithm may then be compared to the known ground truth to obtain the deformation error for each image voxel. Expert drawn contours are also included for several structures in order to provide error statistics for individual organs. Table 1 shows the patient characteristics for each of the virtual phantoms, and Fig. 1 shows an example of one of the phantoms.

**Table 1:** Attributes of patients selected for the development of the virtual phantoms.

*Patient No.*	*Disease Site*	*Stage*	*Gender*	*Fractions Delivered*	*Mean Right Parotid Dose (Gy)*	*Mean Left Parotid Dose (Gy)*	*Initial Weight/End of Treatment Weight (kgs)*	*No. of Days Between Planning and EOT Images*
1	Base of tongue	T2N2bM0	M	35	25.2	25.7	74.8/70.3	60
2	Base of tongue	T2N2cM0	F	35	34.7	23.2	68.0/62.1	56
3	Tonsil	T2N2bM0	M	35	29.4	25.6	96.2/88.5	57
4	Nasopharynx	T1N3M0	F	33	26.7	39.5	65.3/61.2	58
5	Unknown	T0N2aM0	M	35	24.5	29.0	90.3/81.2	57
6	Supraglottic larynx	T1N1M0	M	33	26.1	21.3	95.3/82.6	43
7	Tonsil	T2N2aM0	M	35	14.2	41.7	93.4/86.2	47
8	Tonsil	T2N2aM0	F	35	23.5	28.5	106.1/101.6	48
9	Nasopharynx	T4N2M0	M	33	55.7	48.7	68.0/56.7	59
10	Base of tongue	T0N2aM0	F	35	21.5	23.4	99.8/81.2	68

EOT=end of treatment.

### B. DIR algorithm evaluation

At the time of evaluation, each software package had only one primary algorithm available for DIR represented by the algorithms described below. In order to protect intellectual property, companies that market DIR systems typically do not disclose detailed information about their algorithms. Therefore, each algorithm will be treated as a “black box” for the purposes of this investigation. The virtual phantom image pairs were imported into each system for registration. To avoid an additional confounding variable, an initial rigid registration was not performed before DIR for four out of the five algorithms examined. It was not feasible to disable the initial rigid registration for the Pinnacle algorithm, but this should not substantially affect the results because the phantom image pairs were already well‐aligned in all of the systems before initiating the deformation. For each registration, the SOT dataset was designated as the primary dataset (the target) and the simulated EOT dataset was designated as the secondary dataset (to be deformed to match the target). This arrangement was chosen to mimic an adaptive workflow, in which a treatment plan is recalculated on daily IGRT imaging and the dose is then transferred back to a planning CT for evaluation. The registration procedure for each algorithm is described in the Material & Methods B.1 Algorithm 1: MIM, B.5 Algorithm 5: Eclipse below. After registration, DVFs were exported from each system and compared to the ground‐truth DVFs using MATLAB (MathWorks Inc., Natick, MA). DIR error statistics were calculated for all of the voxels contained within the brainstem, spinal cord, mandible, left parotid, right parotid, and external contours.

#### B.1 Algorithm 1: MIM

Deformable registration was performed using the VoxAlign algorithm incorporated with MIM version 5.6.2 (MIM Software Inc., Cleveland, OH). The VoxAlign algorithm is a constrained, intensity‐based, free‐form DIR algorithm. Because the phantom image pairs were in the same DICOM frame of reference, no initial rigid registration was performed. Following deformable registration, the DIR information was saved and exported from the system as a DICOM Deformable Spatial Registration object. Reg Reveal and Reg Refine (MIM Software Inc.) were implemented recently into MIM as tools that allow the user to view and refine deformable registrations. These tools were not used in this study.

#### B.2 Algorithm 2: VelocityAI

The deformable multipass algorithm of VelocityAI version 2.8.1 (Varian Medical Systems, Palo Alto, CA) was used to register the virtual phantoms. The deformable multipass algorithm is a multiresolution B‐spline algorithm. No initial rigid registration was performed between the image pairs. The DVF data were exported from VelocityAI as a binary deformation field (BDF) file for analysis.

#### B.3 Algorithm 3: RayStation

The hybrid deformable registration algorithm of RayStation version 3.5 (RaySearch Laboratories, Stockholm, Sweden) was used to register the phantoms. The hybrid deformable registration algorithm is an intensity‐based algorithm that can also incorporate ROI constraints. RayStation requires that a rigid registration be performed before initiating a deformable registration. Therefore, the “Set Identity” registration tool was used to mark the initial coordinates of each image pair as aligned. In other words, all initial rigid registration parameters were set to zero. The phantoms' external contours were defined as required, but no other ROIs were specified. A deformation grid of 2.5×2.5×3.0 mm was selected for each case. RaySearch provided a script that enabled the export of the calculated DVFs.

#### B.4 Algorithm 4: Pinnacle

DIR was accomplished using the Dynamic Planning Module of Pinnacle^3^ 9.6 (Philips Healthcare, Fitchburg, WI). The Pinnacle^3^ system used an implementation of the demons algorithm for deformable registration. Before DIR, rigid registration and image preprocessing were performed on the virtual phantom image pairs. DVFs were exported from the software as binary files.

#### B.5 Algorithm 5: Eclipse

The DIR tools in Eclipse version 11 (Varian Medical Systems) were used to register the virtual phantom images. The Eclipse treatment planning system also uses an implementation of the accelerated demons algorithm. Rigid registration was not performed prior to deformable registration. DVF information was exported from the software as a DICOM Deformable Spatial Registration object.

### C. Propagation of dose

As noted previously, the 10 DIREP phantoms were created from real patient data. In addition to imaging, this data included treatment plans which were subsequently used in this study to calculate dose on each EOT dataset. All of the treatment plans were calculated and delivered using the TomoTherapy treatment platform (Accuray Inc., Sunnyvale, CA). This dose was then transferred to the individual SOT datasets via the ground‐truth DVF. Dose was also transferred through the DVFs provided by each DIR algorithm. The difference between the two dose propagation methods (ground truth vs. test) was evaluated in terms of the difference in mean and maximum (max) dose to each organ at risk. The effect of TRE on DVH error was also quantified by calculating the dosimetric difference in the DVH for each organ on a point‐by‐point basis along the DVH. This metric, termed the mean DVH difference $\left(\Delta {\text{DVH}}_{\text{mean}}\right)$, was used along with mean and max dose difference in Pearson correlations with both mean $\left({\text{TRE}}_{\mu }\right)$ and max TRE. Due to the fact that all treatment plans were not prescribed for the same total dose, only the dose as calculated for a single 2 Gy fraction was used for this study. All treatment plans were originally prescribed at this fractionation pattern, as indicated in Table 1. At the time of the writing of this manuscript, the Pinnacle and Eclipse platforms did not perform deformable dose accumulation. They are included here for comparison purposes.

## III. RESULTS

**Figure 2:**
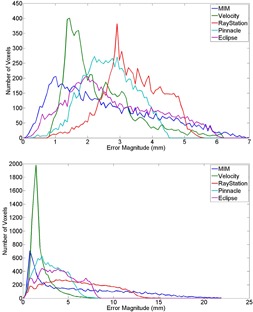
TRE histograms for the left (top) and right (bottom) parotids of Phantom 9. The histograms show the number of voxels in the designated ROI that were deformed with the error specified by the x‐axis for each algorithm. Note the scale differences of each histogram.

Target registration error for each OAR is presented in 2, 3, 4, 5, 6, 7. As shown, the differences between the algorithms are small, although registrations performed using the MIM algorithm did consistently produce lower mean errors. This difference was found to be significant (*t*‐test, df=10,p<0.05) for each OAR, except the right parotid. For this organ, no significance was found between any of the five DIR algorithms. Interestingly, both MIM and RayStation also generated a large misregistration specific to the right parotid of Phantom 9 (${\text{TRE}}_{\mu }>6\;\text{mm}$ and ${\text{TRE}}_{\text{max}}>15\;\text{mm}$ for both algorithms). In order to better visualize this result, histograms of the TRE of the left and right parotid of Phantom 9 are shown in Fig. 2. The long tail of the histogram evident in the right parotid but absent from left parotid indicates the large maximum errors generated by the MIM and RayStation algorithms for this case. Registration error histograms for all of the ROIs and phantoms are available in the Supplementary Materials file available on the JACMP website at www.jacmp.org

**Table 2:** Registration error statistics for all of the voxels contained within the brainstems of the virtual phantoms. Statistics are listed as the mean ±1 SD. The maximum errors are shown in parentheses. All errors are reported in mm.

*Phantom No.*	*MIM*	*Velocity*	*RayStation*	*Pinnacle*	*Eclipse*
1	0.3±0.1 (0.6)	1.8±0.2 (2.3)	0.8±0.5 (1.8)	3.8±2.0 (10.0)	0.9±0.4 (1.9)
2	0.2±0.1 (0.6)	2.0±0.3 (3.0)	1.5±0.8 (2.9)	4.3±1.9 (10.0)	0.7±0.4 (2.1)
3	0.4±0.2 (0.8)	0.7±0.3 (1.5)	1.9±0.6 (3.7)	1.4±0.6 (3.3)	1.2±0.4 (2.1)
4	0.3±0.1 (0.7)	1.2±0.3 (1.9)	1.2±0.1 (1.4)	1.0±0.5 (3.4)	1.1±0.6 (2.7)
5	0.3±0.1 (0.7)	1.0±0.3 (1.8)	2.2±0.8 (4.1)	6.3±2.5 (13.2)	0.7±0.4 (2.5)
6	0.5±0.2 (0.9)	0.7±0.1 (1.2)	1.0±0.2 (1.6)	1.4±0.7 (4.3)	1.0±0.6 (3.2)
7	0.8±0.2 (1.5)	0.9±0.3 (1.6)	0.7±0.3 (1.6)	1.5±0.8 (4.4)	1.3±0.5 (3.1)
8	0.5±0.2 (1.2)	0.9±0.3 (1.7)	1.3±0.4 (2.2)	6.8±3.3 (16.1)	1.5±0.5 (3.0)
9	0.8±0.3 (1.5)	1.7±0.3 (2.6)	3.0±0.5 (4.1)	3.3±1.5 (9.2)	2.1±0.9 (5.0)
10	0.7±0.4 (2.5)	1.1±0.3 (1.9)	0.7±0.3 (1.6)	3.5±2.1 (10.9)	1.0±0.4 (2.5)
Mean	0.5±0.2 (2.5)	1.2±0.5 (3.0)	1.4±0.7 (4.1)	3.3±2.1 (16.1)	1.1±0.4 (5.0)

**Table 3:** Registration error statistics for all of the voxels contained within the spinal cords of the virtual phantoms. Statistics are listed as the mean ±1 SD. The maximum errors are shown in parentheses. All errors are reported in mm.

*Phantom No.*	*MIM*	*Velocity*	*RayStation*	*Pinnacle*	*Eclipse*
1	0.3±0.2 (1.0)	2.2±0.4 (2.9)	0.3±0.2 (1.2)	0.7±0.3 (1.7)	0.7±0.3 (1.7)
2	0.4±0.2 (0.8)	2.1±0.6 (3.4)	0.7±0.2 (1.4)	0.7±0.4 (2.4)	0.9±0.3 (1.8)
3	0.7±0.5 (2.6)	1.9±0.9 (3.8)	2.4±0.9 (4.8)	1.4±0.6 (3.2)	1.4±0.5 (3.0)
4	0.4±0.3 (1.2)	1.1±0.4 (2.0)	0.5±0.2 (1.2)	0.8±0.4 (1.9)	0.9±0.5 (2.5)
5	0.4±0.2 (1.0)	0.8±0.2 (1.3)	0.5±0.2 (1.4)	1.3±0.7 (4.3)	0.9±0.6 (2.9)
6	0.5±0.2 (0.9)	0.7±0.2 (1.5)	2.1±1.2 (4.4)	1.1±0.4 (2.5)	0.8±0.3 (1.7)
7	0.5±0.2 (1.7)	4.7±4.3 (14.8)	1.8±1.6 (7.7)	1.3±0.8 (3.9)	1.9±0.9 (4.2)
8	0.5±0.3 (1.3)	1.1±0.4 (2.6)	0.3±0.2 (1.1)	0.7±0.3 (2.6)	1.0±0.5 (3.8)
9	0.5±0.2 (1.1)	2.0±0.5 (2.8)	0.9±0.5 (3.0)	1.2±0.5 (3.1)	1.2±0.5 (2.9)
10	0.5±0.2 (1.1)	0.9±0.2 (1.6)	0.4±0.2 (1.0)	1.2±0.7 (3.5)	1.2±0.4 (2.4)
Mean	0.5±0.1 (2.6)	1.8±1.2 (14.8)	1.0±0.8 (7.7)	1.0±0.3 (4.3)	1.1±0.3 (4.2)

**Table 4:** Registration error statistics for all of the voxels contained within the mandibles of the virtual phantoms. Statistics are listed as the mean ±1 SD. The maximum errors are shown in parentheses. All errors are reported in mm.

*Phantom No.*	*MIM*	*Velocity*	*RayStation*	*Pinnacle*	*Eclipse*
1	0.5±0.5 (2.4)	2.1±0.5 (3.4)	0.8±0.3 (1.9)	1.0±0.5 (3.0)	1.9±0.9 (4.5)
2	0.7±0.5 (4.7)	1.5±0.7 (4.9)	0.9±0.6 (5.6)	1.0±0.7 (6.2)	1.7±0.7 (7.0)
3	1.2±0.9 (4.9)	1.5±1.0 (4.7)	2.8±1.7 (6.9)	1.2±0.6 (3.0)	1.5±0.6 (4.4)
4	0.6±0.6 (3.1)	1.0±0.4 (2.2)	1.7±1.2 (6.1)	0.9±0.4 (3.0)	1.5±0.6 (4.0)
5	0.7±0.6 (4.8)	0.9±0.4 (2.7)	2.9±2.2 (8.6)	1.2±0.7 (5.3)	1.6±0.9 (5.0)
6	0.9±0.6 (2.7)	1.1±0.4 (2.2)	1.4±1.0 (6.2)	1.1±0.5 (3.5)	1.2±0.5 (3.0)
7	1.1±0.7 (3.5)	1.4±0.4 (2.6)	1.2±0.6 (3.6)	0.9±0.5 (2.8)	1.6±0.6 (3.3)
8	0.7±0.6 (4.9)	1.4±0.7 (4.6)	1.4±0.7 (4.4)	2.4±1.4 (6.4)	3.5±2.0 (9.5)
9	1.5±1.2 (6.3)	1.8±0.8 (5.6)	1.7±1.1 (5.2)	1.3±0.8 (5.6)	2.6±1.4 (7.7)
10	0.6±0.4 (2.5)	1.9±1.0 (5.4)	0.9±0.5 (2.7)	1.4±0.9 (4.8)	3.5±1.5 (7.7)
Mean	0.9±0.3 (6.3)	1.5±0.4 (5.6)	1.6±0.7 (8.6)	1.2±0.4 (6.4)	2.1±0.9 (9.5)

**Table 5:** Registration error statistics for all of the voxels contained within the left parotids of the virtual phantoms. Statistics are listed as the mean ±1 SD. The maximum errors are shown in parentheses. All errors are reported in mm.

*Phantom No.*	*MIM*	*Velocity*	*RayStation*	*Pinnacle*	*Eclipse*
1	1.6±1.6 (7.9)	2.7±0.3 (3.9)	1.8±1.1 (5.0)	1.3±0.6 (3.1)	1.7±1.2 (5.0)
2	0.7±0.5 (3.5)	1.5±0.6 (2.6)	1.7±0.9 (4.5)	2.5±1.1 (6.0)	2.1±1.2 (5.0)
3	1.8±1.8 (10.8)	1.7±1.2 (6.7)	4.5±1.9 (11.8)	2.5±1.3 (9.4)	3.2±2.2 (11.4)
4	0.9±0.7 (3.5)	2.3±1.1 (6.1)	1.1±0.4 (2.9)	2.6±1.3 (5.9)	1.7±0.9 (4.1)
5	1.1±1.2 (7.3)	2.8±1.6 (8.7)	2.6±1.6 (8.8)	2.5±1.2 (7.2)	1.5±0.8 (4.9)
6	0.7±0.7 (4.4)	1.8±0.8 (5.1)	1.3±0.7 (4.2)	1.1±0.5 (3.2)	1.1±0.5 (2.6)
7	0.8±0.4 (2.5)	0.9±0.3 (1.7)	1.1±0.5 (3.1)	0.8±0.4 (2.5)	1.9±0.8 (3.7)
8	1.0±1.1 (6.9)	3.0±1.1 (6.6)	1.5±0.8 (4.3)	1.5±1.1 (6.2)	3.2±1.8 (7.8)
9	2.5±1.5 (7.0)	2.3±1.0 (6.2)	3.4±0.9 (5.5)	2.7±0.8 (4.5)	2.8±1.4 (6.2)
10	0.6±0.6 (4.1)	2.8±0.8 (6.0)	1.3±1.1 (5.7)	1.5±0.9 (5.1)	1.8±1.0 (4.1)
Mean	1.2±0.6 (10.8)	2.2±0.7 (8.7)	2.0±1.1 (11.8)	1.9±0.7 (9.4)	2.1±0.7 (11.4)

**Table 6:** Registration error statistics for all of the voxels contained within the right parotids of the virtual phantoms. Statistics are listed as the mean ±1 SD. The maximum errors are shown in parentheses. All errors are reported in mm.

*Phantom No.*	*MIM*	*Velocity*	*RayStation*	*Pinnacle*	*Eclipse*
1	1.1±1.0 (5.0)	1.9±0.5 (3.6)	1.3±0.7 (3.4)	1.3±0.6 (3.1)	1.4±1.0 (3.9)
2	0.7±0.4 (2.8)	2.1±0.4 (2.8)	1.0±0.6 (2.4)	1.5±0.6 (3.3)	3.2±1.8 (6.7)
3	0.4±0.3 (2.2)	0.9±0.4 (2.5)	1.4±0.6 (2.8)	1.1±0.5 (3.2)	1.1±0.5 (3.3)
4	0.9±0.9 (5.6)	1.3±0.4 (3.1)	2.3±0.6 (4.4)	1.7±1.0 (4.8)	1.3±0.7 (4.2)
5	1.6±1.8 (10.2)	1.6±0.7 (4.6)	3.5±1.5 (8.5)	2.6±1.3 (6.3)	1.3±0.9 (5.1)
6	1.5±1.6 (7.4)	1.6±0.7 (4.0)	2.9±1.1 (5.9)	1.3±0.7 (3.6)	1.3±0.9 (4.5)
7	0.8±0.7 (5.2)	1.3±0.3 (2.3)	1.5±0.5 (3.1)	0.8±0.4 (3.1)	1.7±0.8 (3.9)
8	0.7±0.6 (4.5)	2.4±1.1 (4.6)	2.8±0.9 (5.1)	1.8±0.9 (4.8)	1.6±0.8 (4.8)
9	6.3±5.1 (22.0)	1.9±1.1 (7.9)	6.1±3.2 (15.2)	3.1±1.6 (8.3)	4.0±2.0 (8.5)
10	0.6±0.8 (5.8)	1.2±0.5 (3.0)	0.9±0.7 (3.5)	1.5±0.9 (5.0)	1.6±1.0 (4.5)
Mean	1.5±1.7 (22.0)	1.6±0.5 (7.9)	2.4±1.6 (15.2)	1.7±0.7 (8.3)	1.8±1.0 (8.5)

**Table 7:** Registration error statistics for all of the voxels contained within the external contours of the virtual phantoms. Statistics are listed as the mean ±1 SD. The maximum errors are shown in parentheses. All errors are reported in mm.

*Phantom No.*	*MIM*	*Velocity*	*RayStation*	*Pinnacle*	*Eclipse*
1	1.3±2.1 (28.4)	2.1±0.9 (12.2)	1.9±2.8 (23.0)	2.4±2.8 (18.5)	2.6±2.7 (20.3)
2	0.9±1.1 (10.9)	2.1±1.0 (15.1)	2.3±1.8 (13.7)	2.7±3.0 (20.4)	2.3±2.0 (15.4)
3	1.5±2.3 (22.1)	1.7±1.2 (10.7)	4.3±3.4 (20.7)	2.5±2.3 (17.1)	2.2±2.2 (18.5)
4	0.9±1.1 (13.9)	1.5±0.9 (10.9)	1.6±1.4 (16.9)	1.3±1.1 (15.2)	2.2±1.8 (12.4)
5	1.6±2.0 (18.9)	2.2±1.9 (20.7)	3.4±2.7 (30.5)	4.1±3.9 (23.9)	2.6±2.6 (25.7)
6	1.2±1.9 (25.5)	1.3±0.9 (15.7)	2.4±1.9 (19.1)	2.1±1.9 (16.3)	2.3±2.3 (16.8)
7	1.1±1.6 (19.2)	2.0±2.0 (23.3)	3.0±3.4 (19.8)	1.7±1.8 (16.2)	2.5±1.8 (17.8)
8	1.4±1.5 (18.8)	1.9±1.2 (14.1)	3.2±2.4 (23.7)	3.1±3.0 (22.6)	3.2±2.5 (19.2)
9	1.8±2.5 (29.8)	2.1±1.1 (15.4)	3.1±2.6 (26.1)	3.3±2.8 (24.1)	2.9±2.2 (16.1)
10	2.2±3.5 (31.5)	2.4±1.8 (20.2)	4.1±5.2 (36.8)	3.4±3.9 (35.0)	2.7±2.0 (22.8)
Mean	1.4±0.4 (31.5)	1.9±0.3 (23.3)	2.9±0.9 (36.8)	2.7±0.8 (35.0)	2.5±0.3 (25.7)

**Figure 3:**
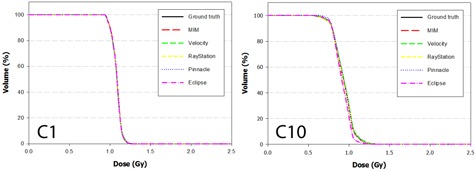
DVH curves of the spinal cord for Phantom 1 and Phantom 10 after dose has been propagated from the EOT to SOT dataset through the ground‐truth and test DVFs. Phantom 1 showed the least disagreement overall with the ground‐truth DVH, while Phantom 10 showed the most disagreement.

**Figure 4:**
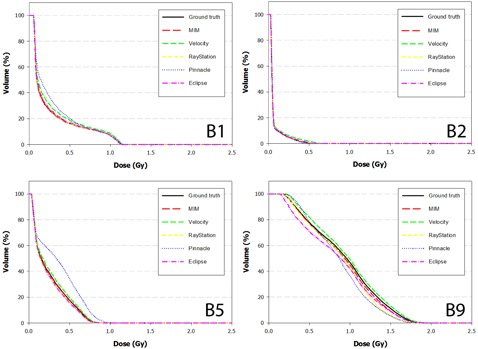
DVH curves of the brainstem for Phantoms 1, 2, 5, and 9 after dose has been propagated from the EOT to SOT dataset through the ground‐truth and test DVFs. Note the strong convergence with the ground truth for Phantom 2. In this case, the dose distribution did not include the nasopharynx, increasing the distance between the brainstem and the high dose regions of the treatment plan.

**Figure 5:**
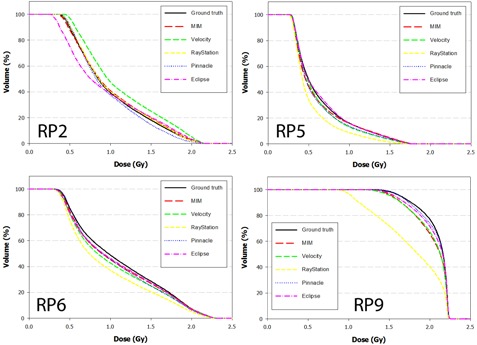
DVH curves of the right parotid for Phantoms 2, 5, 6, and 9 after dose has been propagated from the EOT to SOT dataset through the ground‐truth and test DVFs. Note disagreement for Phantom 9 when dose is transferred using the RayStation algorithm. In this case, the mean target registration error was equal to 6.1 mm and a high dose gradient directly traversed the right parotid.

**Figure 6:**
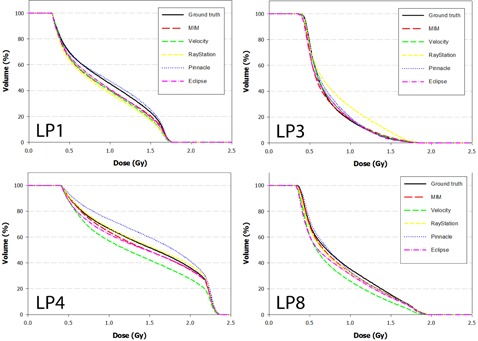
DVH curves of the left parotid for Phantoms 1, 3, 4, and 8 after dose has been propagated from the EOT to SOT dataset through the ground‐truth and test DVFs. Note disagreement for difference between the Velocity and Pinnacle algorithms for Phantom 4. This occurs because the DVF for each algorithm mapped voxels in opposite directions away from the ground truth.

**Figure 7:**
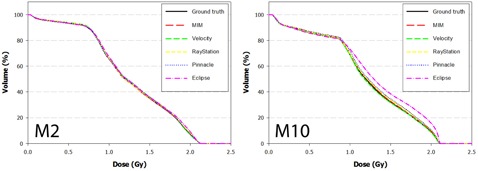
DVH curves of the mandible for Phantoms 2 and 10 after dose has been propagated from the EOT to SOT dataset through the ground‐truth and test DVFs.

**Figure 8:**
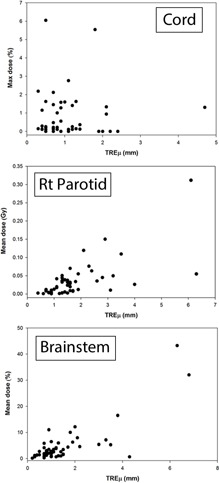
Correlation between mean target registration error and dosimetric error for the spinal cord, right parotid, and brainstem. Pearson correlation coefficients can be found for the distributions in Table 8.

The DVH results from the dose propagation study are shown in Fig. 3–7. Starting with the spinal cord, Fig. 3 illustrates the best (Phantom 1) and worst (Phantom 10) case scenarios as scored by $\Delta {\text{DVH}}_{\text{mean}}$. It is evident from the graph that TRE had little effect on the DVH for this structure. This is further supported in the Pearson correlations (Table 8 and Fig. 8) which show several dosimetric parameters having only a weak to very weak correlation with both ${\text{TRE}}_{\mu }$ and ${\text{TRE}}_{\text{max}}$. In direct comparison, the brainstem shows a strong to very strong correlation with ${\text{TRE}}_{\mu }$ and a moderate correlation with ${\text{TRE}}_{\text{max}}$. Four DVHs of the brainstem are highlighted in Fig. 4, showing both similarity and divergence amongst the algorithms for different cases. The remaining OARs show a moderate to strong correlation with ${\text{TRE}}_{\mu }$ and a very weak correlation with ${\text{TRE}}_{\text{max}}$. The previously mentioned misregistration in the right parotid of Phantom 9 is shown in Fig. 5 (bottom right). Interestingly, while both MIM and RayStation produced similar mean errors (∼6 mm), clearly these errors did not affect the DVH in the same way. Another interesting case is that of the left parotid of Phantom 4. In this instance, there is a large deviation in both directions away from the ground‐truth DVH. The net effect on the mean dose to the left parotid is a 10.4% increase when using Velocity compared to an 8% decrease when using Pinnacle. This was the largest discrepancy encountered in this study amongst the five commercial algorithms.

**Table 8:** Pearson correlation coefficients for several different combinations of target registration error $\left(P_{1}\right)$ and dosimetric error $\left(P_{2}\right)$.

$\left(P_{1}\right)$	${\text{TRE}}_{\mu }\;(\text{mm})$	${\text{TRE}}_{\mu }\;(\text{mm})$	${\text{TRE}}_{\mu }\;(\text{mm})$	${\text{TRE}}_{\mu }\;(\text{mm})$	${\text{TRE}}_{\text{max}}\;(\text{mm})$
$\left(P_{2}\right)$	$D_{\mu }$ *(%)*	$D_{\mu }$ *(Gy)*	*DVH (Gy)*	$D_{\text{max}}$ *(Gy)*	$D_{\text{max}}$ *(Gy)*
Brainstem	0.807	0.717	0.752	0.585	0.470
Mandible	0.672	0.705	0.637	0.211	0.122
Parotid Rt	0.531	0.668	0.647	0.161	0.010
Parotid Lt	0.523	0.484	0.449	0.194	0.140
Cord	0.265	0.286	0.273	0.188	0.132
External	0.262	0.220	0.404	0.320	0.192

## IV. DISCUSSION

Benchmarking is a process in which standardized tests are used to assess system performance. In many applications the results are strictly used for intercomparison (i.e., which processor computes the fastest, which business model is more profitable, etc.). In this application, however, the primary purpose is less about the intercomparison of algorithms and more about the establishment of clinically relevant baseline data. This data, as presented in 2, 3, 4, 5, 6, 7, may be used in a number of ways. First, users of one of these commercial algorithms may download the 10 head and neck datasets and run them as part of their commissioning process, using the benchmarks for comparison. Additionally, during upgrades to newer software versions, the cases may be rerun as a way of maintaining self‐consistency, as discussed by AAPM's Task Group 53.^(14)^ As an example, the large registration errors in the brainstem seen when utilizing the Pinnacle algorithm (Table 2) were reported to the vendor and have reportedly been fixed in current updates to their software. This can be verified using the proposed methodology and will be done in future work.

The benchmark data also provide users with a general idea of the magnitude and variation in TRE for the specific use case adopted in this study — head and neck dose adaptation. As an example, the mean registration error for the spinal cord using the MIM algorithm was 0.5 ± 0.1 mm with an associated standard error of 0.03 mm (n=10). These statistics provide high confidence that the TRE for an individual deformable registration using the MIM algorithm under similar conditions will be less than 1 mm for this structure. Conversely, in looking at the right parotid when applying the RayStation algorithm, the mean error was 2.4±1.6 mm, with an associated standard error of 0.5 mm. Users in this situation can thus expect higher variability when considering an individual case where the mean error may range between 2–3 mm.

In comparison to other studies, Varadhan et al.^(13)^ and Nie et al.^(15)^ used ImSimQA to create virtual H&N phantoms and evaluate DIR. The Varadhan study reported contour comparison metrics between contours deformed using the ground‐truth DVF and contours deformed using two different DIR algorithms. While these metrics are useful for contour comparison studies, they are of limited use for deformable dose accumulation or other DIR applications, and would be difficult to compare to the data presented here. This fact highlights the need for consistent metrics and datasets to compare DIR algorithms. The Nie study used a single virtual H&N phantom, along with phantoms for other treatment sites, to compare the DVFs generated by MIM 5.4.7 and VelocityAI 2.2.1 to the ground‐truth provided by the ImSimQA DVF. The MIM algorithm resulted in 24.2% of voxels having errors greater than 2 mm, 14.6% greater than 3 mm, and only 7% greater than 5 mm. The VelocityAI algorithm resulted in 29.8% of voxels having errors greater than 2 mm, 5.1% greater than 3 mm, and only 0.1% greater than 5 mm. These results are consistent with some of the phantom data reported in this study. However, the differences that we observed between phantoms emphasize the need to evaluate multiple cases for comprehensive DIR QA.

**Figure 9:**
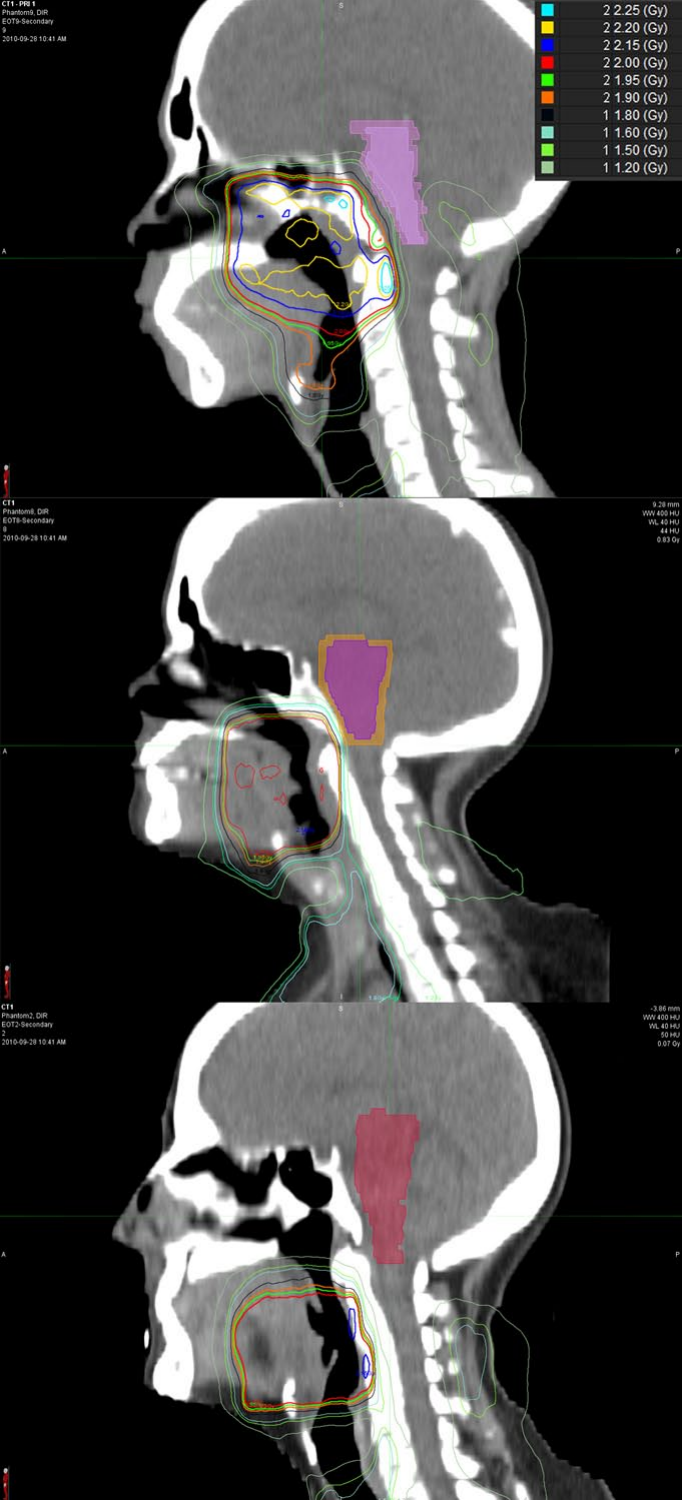
Dose distribution shown for Phantom 2 (bottom), Phantom 8 (middle), and Phantom 9 (top). In comparing the DVH and TRE data for these patients, it is evident that the distance between the brainstem and dose gradient plays a large role in the sensitivity to dosimetric error caused by TRE itself.

To this point, it is evident that, on an individual basis, H&N DIR can result in large spatial errors which may be difficult to detect. All of the commercial systems generated registrations that could be considered “failures” in the sense that they produced a ${\text{TRE}}_{\mu }$ more than two standard deviations (SDs) away from the overall system‐independent average (${\text{TRE}}_{\text{avg}}$) for selected ROIs. The effects of these failures on a given DVH are not easy to predict. As noted in the results, the strongest correlations between TRE and dosimetric error were in the brainstem. In looking at the three instances where the ${\text{TRE}}_{\mu }$ qualified as a failure (Pinnacle – Phantoms 2, 5, and 8), two out of the three cases also generated large dosimetric errors (Phantoms 5 and 8), while the third case did not (Phantom 2). In viewing the dose distributions for these phantoms it is clear why this occurred. As seen in Fig. 9, the treatment plan for Phantom 2 did not encompass the nasopharynx, which resulted in a larger distance between the brainstem and the high‐dose regions of the treatment plan. Also shown in the figure are dose distributions for Phantoms 8 and 9, which can be compared with the DVH data from Fig. 4 and the TRE data from Table 2.

Only the single large error from the Pinnacle algorithm (6.8 mm) appears to affect the DVH for Phantom 8, while smaller errors from all the algorithms (0.8–3.3 mm) produced divergence in the DVH for Phantom 9. The common trend amongst the three phantoms is that the closer the brainstem is to the dose gradient, the more sensitive the brainstem becomes to dosimetric error caused by TRE.

**Figure 10:**
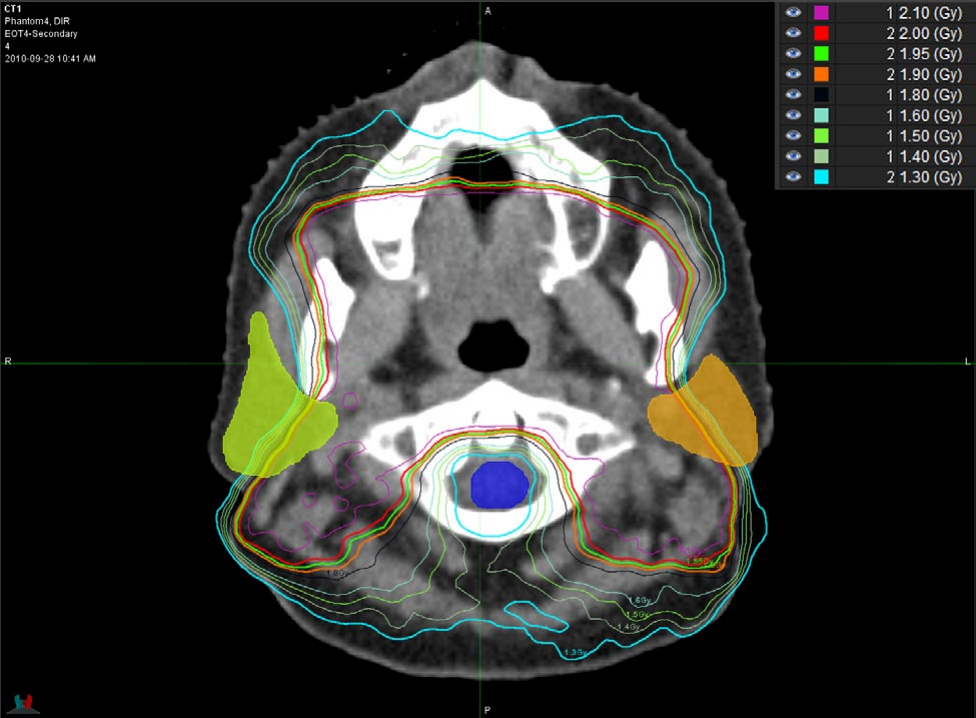
Dose distribution shown for Phantom 4. Note the steep dose gradients which traverse the parotids but avoid the cord. Large differences were seen in the DVH for each parotid, but little difference was seen when considering the spinal cord.

These cases illustrate the difficulty in trying to generalize the correlation between TRE and DVH error. Clearly, the correlation depends upon not only the magnitude of the registration error, but also the dose distribution itself, including the slope of nearby dose gradients and the distance to critical structures. This is highlighted further in Fig. 10 where sharp dose gradients traverse both parotids, but avoid the cord of Phantom 4. In this case, large differences were seen amongst the five algorithms when viewing the DVH for each parotid, but little difference was seen when considering the spinal cord. While ${\text{TRE}}_{\text{avg}}$ for the cord was lower compared to both parotids for this phantom, the trend also held true for other cases such as Phantom 7 where ${\text{TRE}}_{\text{avg}}$ for the cord was nearly double that of the parotids. This is further reflected in the Pearson correlations for the cord which show only weak to very weak correlation with ${\text{TRE}}_{\mu }$.

This is likely attributed to the fact that H&N plans uniformly avoid dosing the cord by applying margins to this structure. The margin increases the distance between high dose gradients and the true cord, thus mitigating the impact of target registration error.

**Figure 11:**
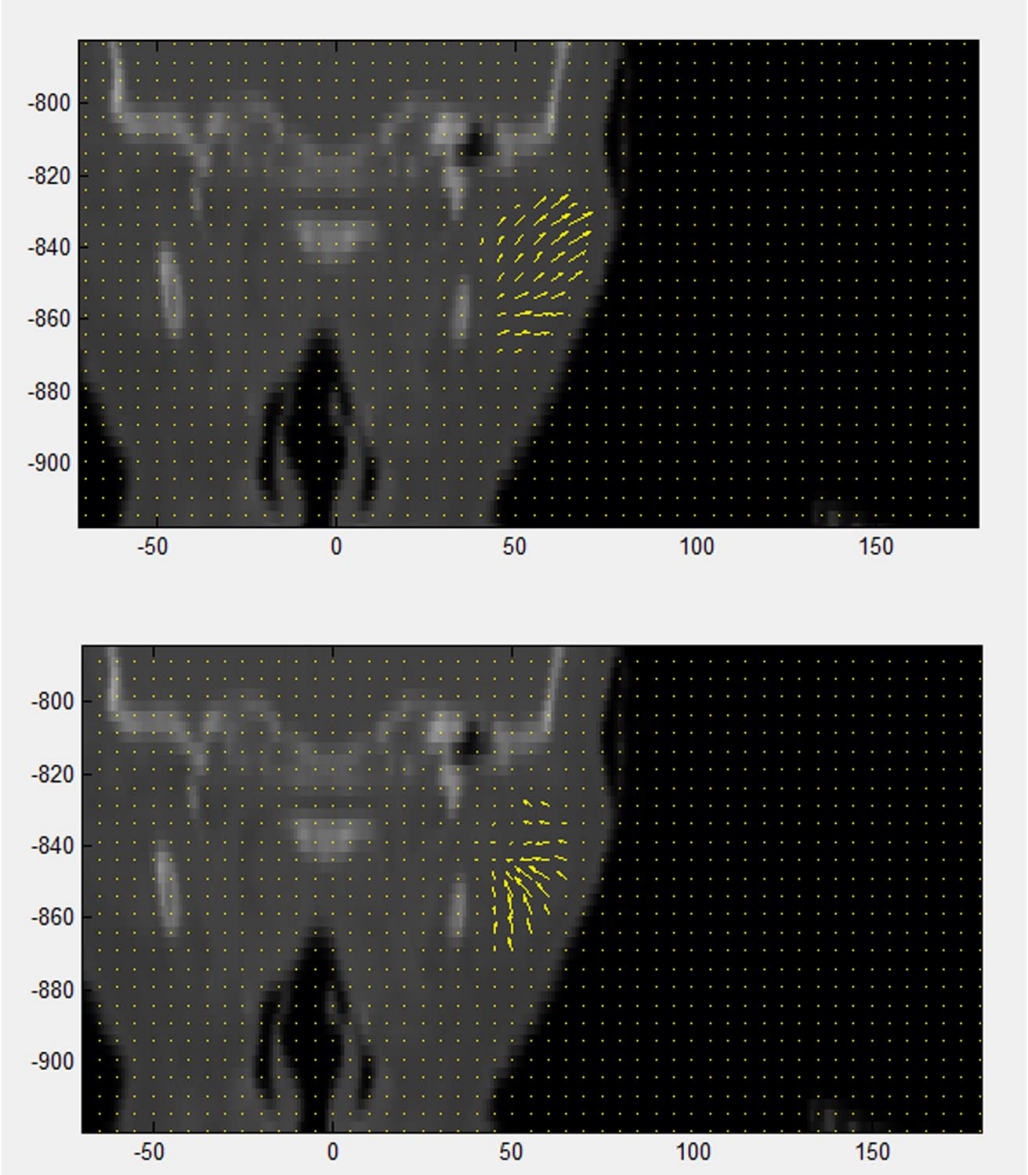
Coronal view of Phantom 4 showing the DVF within the left parotid as determined by the Pinnacle (below) and Velocity (above) algorithms. Note the opposite direction of the vector fields towards and away from the high‐dose regions of the plan, which is located medially.

For other structures, the direction of the error also played a significant role. The previously mentioned left parotid of Phantom 4 is a prime example, where the DVF produced by the Velocity algorithm erred towards the low‐dose region while the DVF produced by the Pinnacle algorithm erred towards the high‐dose region (Fig. 11). This led to DVH curves which were shifted in opposite directions (Fig. 6). Without information on both the direction of the error and the distance to the dose gradient, this result would be difficult to contextualize. It is thus important to note that the benchmark data published in this study should be interpreted for individual cases in combination with *a priori* knowledge of the dose distribution, keeping in mind all the factors that attribute to dosimetric uncertainty.

It should also be noted that the data obtained in this work from the virtual phantoms is likely only applicable to cases involving the same treatment site, imaging modality, and magnitude of anatomical changes. For these phantoms, that would include kVCT images acquired from H&N patients with appropriate immobilization over a single course of treatment. Our experience has shown that deformation algorithms may behave differently depending on these factors. The virtual phantoms presented in this study also have inherent limitations. All of the complexities of the deformation of the human body during a radiotherapy course would be difficult to model. For example, these phantoms do not model sliding interfaces as might be found in the expansion or contraction of the lungs. They also do not model cavities that appear in one image but not the other.

## V. CONCLUSIONS

In this work, five commercial deformation algorithms have been benchmarked using a set of 10 computation H&N phantoms. The benchmarks have been presented using the error metric TRE, which provides a general assessment of DIR performance. The benchmarks can be used during the commissioning and QA process to help users validate their systems. A dose adaptive strategy was also assessed, whereby TRE was correlated with DVH error. Several factors influenced the results including the magnitude and direction of the registration error, the slope of the dose distribution, and the distance to critical structures. In assessing clinical cases, the latter two pieces of information will be known whereas the former must be estimated based on case studies such as this one. When well‐defined trends exist, such as shown for the spinal cord, the DIR for a given structure can be utilized with high confidence. In other instances caution is warranted. For these scenarios, strategies which include DIR uncertainty into the dose adaptive process should be considered.

## ACKNOWLEDGMENTS

The authors would like to thank each of the vendors for their support in completing this study. This work was partially funded by a grant from Accuray, Inc.

## COPYRIGHT

This work is licensed under a Creative Commons Attribution 4.0 International License.

## Supporting information

Supplementary Material

Supplementary Material

Supplementary Material

Supplementary Material
