# Supplementary figures and images for: Benchmarking of five commercial deformable image registration algorithms for head and neck patients
Source: J Appl Clin Med Phys. 2016 May 8;17(3):25–40. doi: 10.1120/jacmp.v17i3.5735 (PMC5690934; doi:10.1120/jacmp.v17i3.5735)

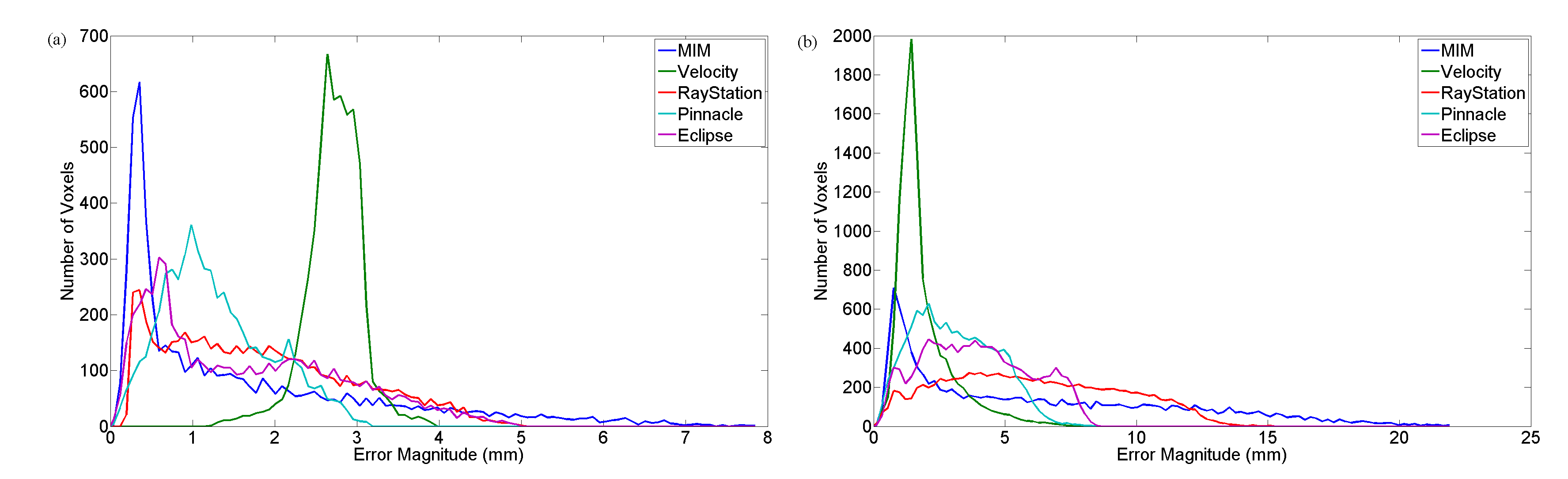

Supplement: Supplementary file 1 — Supplementary Material [file ACM2-17-025-s001.png]

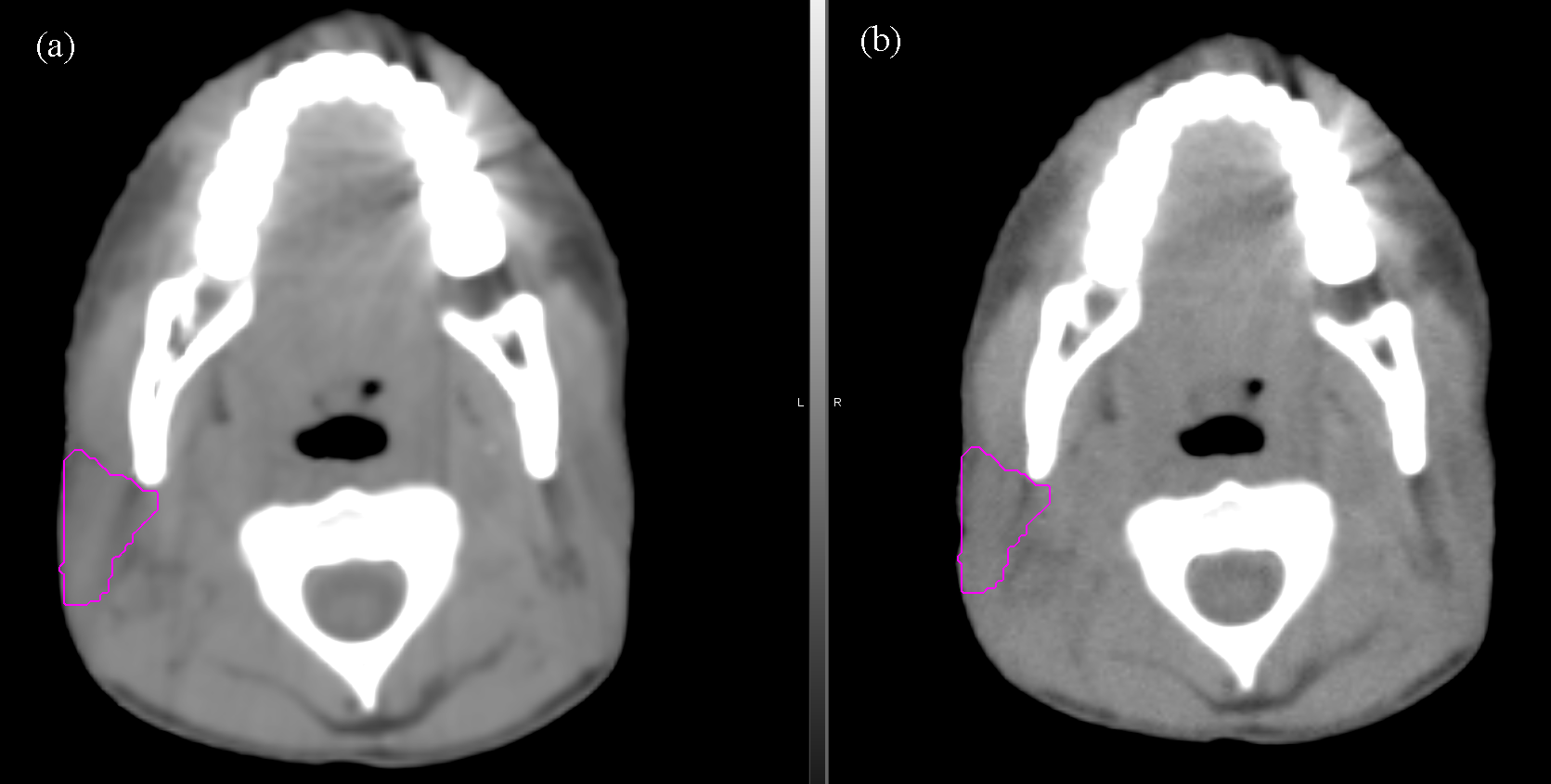

Supplement: Supplementary file 2 — Supplementary Material [file ACM2-17-025-s002.png]

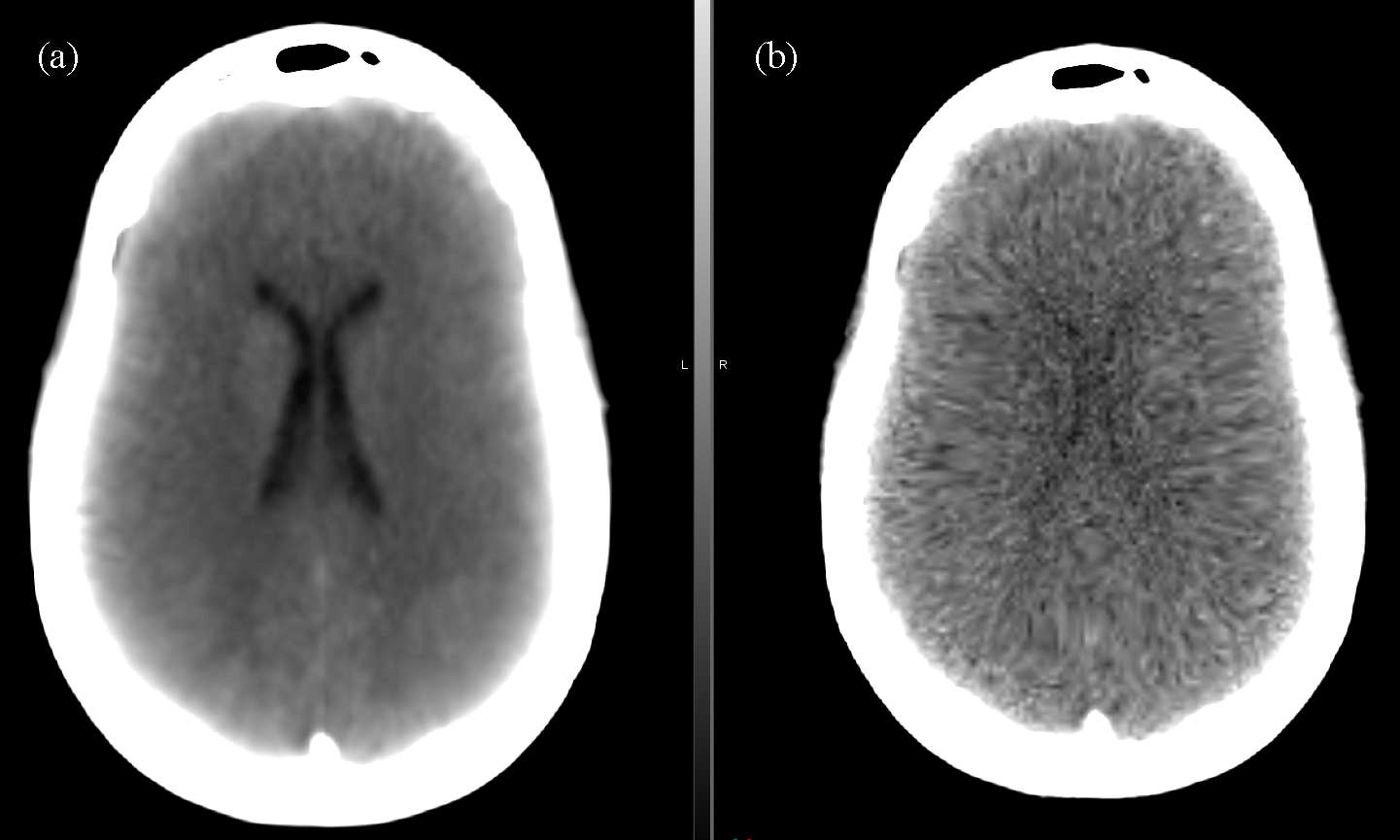

Supplement: Supplementary file 3 — Supplementary Material [file ACM2-17-025-s003.png]

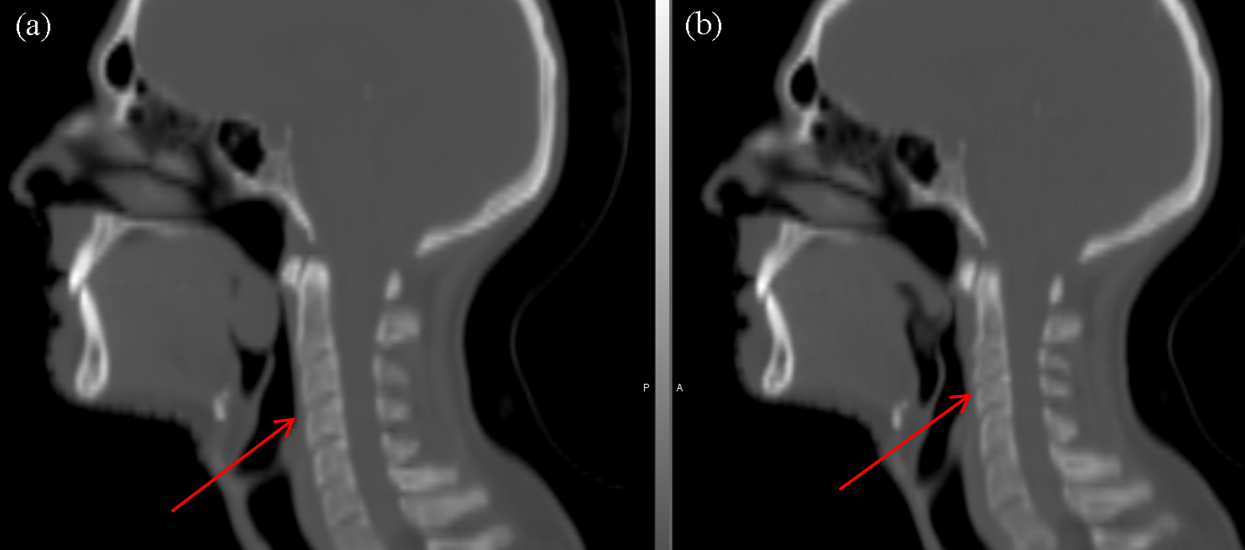

Supplement: Supplementary file 4 — Supplementary Material [file ACM2-17-025-s004.png]
